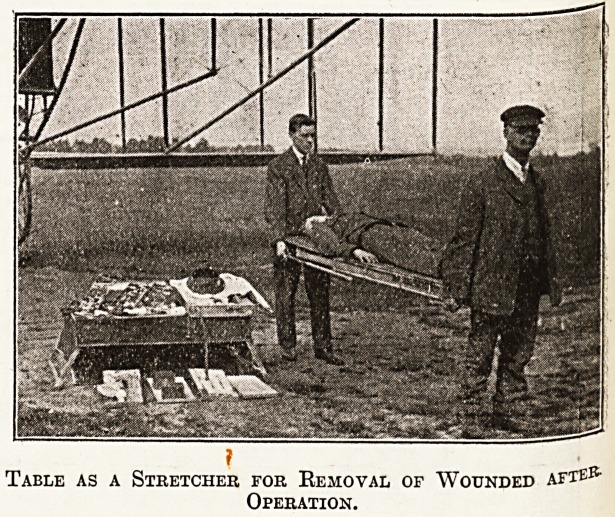# Probable Uses of Aerial Transport to the Medical Profession

**Published:** 1913-08-23

**Authors:** J. D. F. Donegan

**Affiliations:** Royal Army Medical Corps.


### PROBABLE USES OF AERIAL TRANSPORT TO THE MEDICAL PROFESSION.

By Lieut.-Colonel J. D. F. DONEGAN, Royal Army Medical Corps.

Perhaps I may be considered an optimist on the question of uses of aerial transport to the medical profession, and in fact to humanity generally, but so I must remain, waiting patiently until my anticipations are accomplished, though meeting at times with disappointments no doubt. In every scientific branch of study there are people fully imbued with the opinion that something or other connected with an enterprise in the early stage can never be done. Because their exact desire cannot be complied with immediately, every suggestion connected with it appears to them foolish or ridiculous. As regards aerial navigation I should advise those who are only too willing to express adverse comments to be very guarded in their prognosis unless they have no objection to alter their opinions in the face of absolute proof of their mistakes.

Any profession which improves day by day and proves itself of utility to the community at large is often described as progressing in leaps and bounds. In aerial navigation the leaping and bounding stage is over. But a few years ago every one was surprised and satisfied with a flying machine which started on rails and kept in the air for about 150 yards. In a little under four years from that date a monoplane flew 830 miles in a day—that is to say, further than any other mechanically driven contrivance has ever travelled in the same time.

Table Closed up for Transport on Biplane, etc.

Now that that has been done no one can shut their eyes to the future of aerial navigation.

The late Lord Lister introduced antiseptic surgery with no little opposition on the part of his professional confrères some forty-odd years ago. Does anyone object to it to-day? If medicine and surgery had progressed with the same rapidity as aviation, septic poisoning and all contagious diseases would be

Table on Petrol Tins, Showing Surgical Contents on Aerial Cradle.

things of the past, for the simple reason that no septic or infectious germ would be found on the globe. People say: "But flying is so dangerous; everyone who goes in for it gets killed." I don't think I exaggerate when I say that in England, France, Germany, Italy, Russia, and America there are nearly three thousand men in the air every day of the week. When nothing happens nothing is heard, but should an accident occur it is well advertised in the daily newspapers. I do not presume to say that there is not an element of danger, for so there is and so there will continue to be, but care and knowledge go a long way towards preventing mishaps. Carelessness or neglect are likely to be severely punished, but does not the same argument hold good in our own profession? There is danger in the administration of an anæsthetic, and in the performance of abdominal resections; but the respective experts see far less prospects of undesirable consequences than the patient, who generally makes his will and puts his house in order before trusting himself to the surgeon.

The administration of an anæsthetic may be followed by bad results; wounds on occasion will become septic; but our profession would not tolerate the suggestion of discarding anæsthetics or discontinuing surgical operations because there is the remote probability of fatal results. Already an aeroplane has been used for elopement purposes, and a man has flown from France to England with his grandmother as a passenger. In addition to this the Central Flying School has a record of flying 100,600 miles without a serious accident, which I think anyone will admit is tolerably satisfactory.

On the 23rd ult. I had the honour of being listened to by the members of the Ambulance

Section of the British Medical Association at their Brighton meeting when I lectured on this subject. I endeavoured then to find uses for nearly all aerial machines as far as the Medical Service was concerned, even to using small balloons pump inflated and lit with a small dry-cell battery at night-time to designate the localities of our hospitals in towns and on lines of communication in preference to our present method.

I stated my belief that in a short time airships would replace ambulance trains for removing sitting-

Table on Low Level for Reduction of Dislocations or for Use in Shell-Proof Trenches.

up cases, and described the work with (the late) Colonel Cody's new 100-horse-power machine, with which experiments have been tried. I pointed out some defects in the present proposal of leaving cases unfit to be moved to Red Cross Societies, and tried to show that the Societies on many occasions would not be able to cope with serious operations, whereas with Colonel Cody's biplane the assistance of three medical officers would be obtained, not alone from the field, but from hospitals on the lines of communication. Distances which would otherwise take days can be covered by aeroplane in a few hours. I also produced an invention of my own, an aluminium portable operating table made by Arnold and Sons, instrument makers, Giltspur Street, who hold the patent rights, holding enough surgical equipment and instruments to do fifteen or twenty operations, measuring when folded up 22 by 42 inches, and capable of transport on a biplane with a pilot, operator, assistant, and anæsthetist. The table fits on the aeroplane and has to be very compact. All surgical requirements are carried in the table, which can also be used as a stretcher. It is particularly strong and waterproof, and it is provided with modern elevations. The idea is to improve our present Army medical system by which five panniers have to be opened to obtain what is compressed into this table. The space set free in the present panniers by the removal of equipment carried in the table is of course valuable, as can be understood. I have had numerous letters on this subject, and many interested persons ask many questions. Some want to know if I advocate that officers of the Royal Army Medical Corps should all hold flying certificates so as to be able to act as pilots. As time goes on I do not see why a medical practitioner should not pilot his own machine if he so desires, just as he often drives his own motor-car; but I certainly do not advocate his so doing. The careful piloting of an aeroplane requires complete concentration of ideas on one's employment while in flight. If anyone flew a machine, say, a distance of 160 miles, then did a serious surgical operation (or perhaps two), then flew back, and concluded by riding an untrained four-year-old from the aerodrome to his residence he would have had enough nerve stretching and mental anxiety for one day.

The next thing to consider is how aerial machines can be utilised for medical purposes. Personally, were a dentist to tell me that he had ideas of how refractory lower molars could be shifted by the use of an aerial contrivance, I must say I would not condemn it off-hand; I would wait and see. At the present time there is no quicker means of providing urgent medical or surgical assistance than by aerial transport, as the journey can proceed regardless of roads, hedges, ditches, rivers, lakes, or mountains. It may be urged that this means of travelling cannot be used in high winds or at night-time. Even shipping is held up at times by the elements; and as regards night flying, the difficulty rests in landing and seeing where there should happen to be suitable landing ground. Already a form of explosive torch has been invented, which, when dropped from an aeroplane, gives the pilot enough light to see where he is descending, and by its use he can discriminate between water, roofs of houses, trees, or tolerably open ground. I, of course, refer to the future, but I see no reason why aerial trans-

Table as a Stretcher for Removal of Wounded after Operation.

port should not be used for the removal of the injured.

### A Use for Hospital Roofs.

At present a form of wire mooring has been invented which in a way enables a flying machine to be independent of the ground. It certainly is not outside the range of possibility to imagine the roofs of our hospitals fitted with such contrivances and to picture the sick and wounded being conveyed by air and put into their respective wards, even through the windows. The suggestion may appear ridiculous, but ten years ago the suggestion that a man could fly 830 miles in a day would have been considered infinitely more so. Nowadays the open-air treatment for consumption is an accepted fact. Already with the revolving-cone system of anchorage an airship can remain in the air like a ship at anchor. Why should not this means of obtaining the very best of fresh air for incipient tuberculous cases be tried as years roll on?

If in the future we can depend on aerial transport for a safe, rapid, and comfortable means of conveyance of members of our profession, and even of the sick and incapacitated, surely it will be enough. We can hardly expect it to do the hospital washing, though it may be used for drying purposes. I think it behoves our profession and all professions to consider how aerial transport will affect their prospects and to lay their proposals before aerial experts, as they have quite enough to do in their own branch, without attending to outside sources. If a farmer considers that a flying machine can be used for dragging a plough, it is he who should take action. He cannot expect the flying expert to do so, but once the suggestion is brought to his notice he will, I am sure, give his opinion on its feasibility. I do not imagine that my proposals will be carried *nem. con.*, but to those who hold different opinions I most respectfully say that, in spite of ridicule, opposition, or abhorrence, flying-machines, like motor-cars and motor-cycles, have come to stay.

### Colonel Cody's Death.

Since this article was written, one of our finest aerial experts has met with a sudden and untimely end. I am perfectly sure that the accident which resulted in the death of the late Colonel Cody could not have been averted by human hand. According to the evidence available his engines, which, up to date have been the cause of so many accidents, were actually going and the machine flying after the gallant pilot and his passenger had been thrown out.

No doubt in time we shall know what was the cause, but a structural defect in either wood or iron work of this nature could not be detected, even with the most careful examination. Professionally we understand how an operation can be entirely satisfactory and yet the patient may die; but to the man in the street the operation in question is looked on as a failure. The same remark may well be applied to Colonel Cody's machine, which in his own last words he described as being as "steady as a rock."

In conclusion, it may interest many of our profession to know that the late Colonel Cody intended after the all-round Britain race was over to devote his machine to ambulance work and to carry out lengthy experiments, even going as far as try to rig up contrivances to carry helpless lying-down cases on the wings. His ambition was to be of assistance to the wounded soldier and humanity generally. So even we as a profession have suffered a loss by his sudden and noble death. Still we can only work on. There was no use in waiting for the unsinkable ship before we started our experiments, for had we done so as a nation we certainly should not be the maritime Power we are to-day.

## Figures and Tables

**Figure f1:**
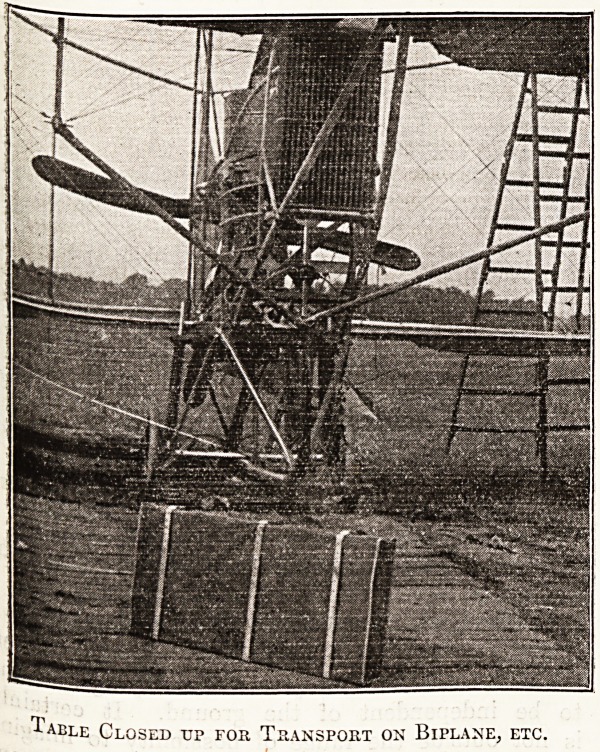


**Figure f2:**
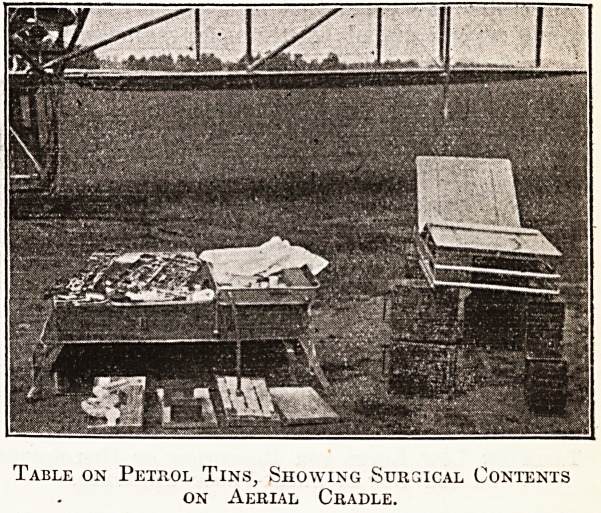


**Figure f3:**
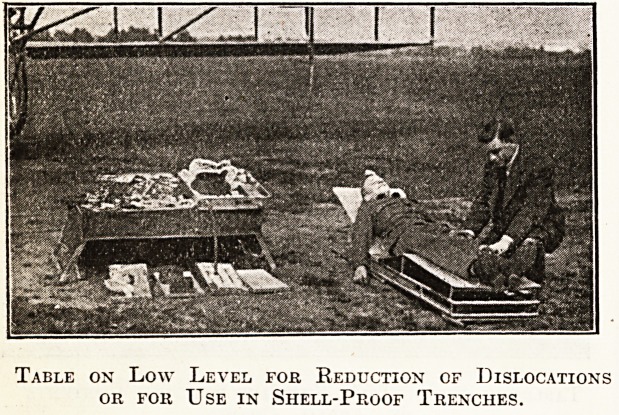


**Figure f4:**